# Research on liquidity and valuation risk assessment of art financial assets based on deep learning

**DOI:** 10.3389/frai.2026.1946060

**Published:** 2026-09-24

**Authors:** Ziwen Jiang, Jingzhou Zhao, Jiamin Yao, Zehao Bian

**Affiliations:** 1 XuBeihong Art Academy, Shanghai Maritime University, Shanghai, China; 2 College of Information Engineering, Shanghai Maritime University, Shanghai, China

**Keywords:** art finance, asset pricing, liquidity risk, multimodal deep learning, valuation risk

## Abstract

Art is increasingly recognized as an alternative asset class in wealth management. However, its non-standardized nature renders it difficult to treat on a par with conventional financial assets. Valuation risk arises from subjective pricing and expert bias, while liquidity risk is manifested in high auction failure rates. These challenges are difficult to address adequately with traditional linear econometric models. This study proposes a multimodal deep learning framework that integrates painting images, historical transaction data, artist reputation, and macroeconomic indicators. A ResNet-50 network, initialized on ImageNet and subsequently adapted to the artwork domain, serves as the visual encoder, while a multi-layer perceptron processes structured tabular variables; the two representations are combined in a fusion layer. The framework simultaneously performs two risk-assessment tasks: valuation deviation regression and unsold-lot binary classification. The model is estimated on 96,314 modern and contemporary auction lots offered between 2000 and 2023, of which 27.8% went unsold, using a strictly chronological training, validation, and test partition. The empirical results demonstrate that the proposed multimodal framework outperforms traditional benchmarks, including ordinary least squares (OLS), logistic regression, and random forests. The model achieves an Area Under the Curve (AUC) of 0.891 for liquidity risk prediction. It reduces the Root Mean Squared Error (RMSE) for valuation deviation by 32.9% compared to OLS. Ablation studies confirm that visual embeddings provide substantial incremental information beyond what can be attributed to increased model complexity alone, and permutation importance combined with linear probing shows that the visual representation encodes stylistic period, subject type, chromatic intensity, and compositional complexity—the properties through which visual content is theorized to affect market outcomes. Robustness checks further delineate the model's performance boundaries under macroeconomic shocks. This study translates the aesthetic heterogeneity of non-standard assets into quantifiable risk signals. It offers financial institutions a data-calibrated basis for adjusting dynamic loan-to-value ratios and assists art funds in optimizing portfolio allocation.

## 1 Introduction

In recent years, art has been transitioning from a collector's object to an alternative asset class in wealth management. Art Basel and UBS report that global art sales reached approximately $57.5 billion in 2024, a 3% increase in volume despite a 12% year-on-year decline in value, and that high-net-worth collectors allocated an average of 20% of their wealth to art in 2025, up from 15% in the previous year (McAndrew, 2025a,b). This expanding demand has not, however, eliminated the fundamental barriers to the financialization of art. Compared with standardized financial assets, art prices are simultaneously influenced by artist reputation, medium, size, provenance, aesthetic preferences, and market sentiment, and trading is characterized by low frequency, discontinuity, and sample selection, rendering return measurement and risk identification susceptible to bias (Korteweg et al., 2016).

Valuation risk and liquidity risk are two of the most critical constraints. Valuation risk stems from the absence of uniform pricing standards; auction house estimates, while informative, may be distorted by commission incentives, anchoring effects, and expert bias. Aubry et al. (2023) construct a neural network to predict auction prices from visual and non-visual features, and find that the higher the machine valuation relative to the auction house's estimate, the higher the sale-price-to-estimate ratio and the lower the abortive rate, suggesting that traditional expert valuation is not fully reliable. Liquidity risk manifests in the difficulty of selling works at reasonable prices when needed; once an auction fails, the price discovery process is interrupted, and the financial utility of artworks as collateral or portfolio assets is correspondingly diminished.

Established studies have largely relied on hedonic price models, repeat-sales indices, or linear regressions, which handle structured variables adequately but struggle to capture non-linear relationships between aesthetic attributes and market outcomes. Compositional, chromatic, brushwork, and stylistic features embedded in painting images carry substantial information that is difficult to encode through manually defined variables. Prior work shows that color and mood can significantly affect the auction value of paintings (Ma et al., 2022), and that multimodal models can fuse image features extracted by convolutional neural networks with structured transaction data (Indrawan et al., 2023). Further, Mei et al. (2025) find that visual embeddings make independent valuation contributions for first-time-to-market works that lack historical price anchors. Meanwhile, the inconsistent performance of tabular machine learning models in art auction prediction suggests that a purely structured-data framework remains insufficient (Mauer and Paszkiel, 2024).

The economic reason why visual content should carry financial information is that, in the art market, the object itself is the security. Three channels connect what a painting looks like to how it trades. The first is a *demand-breadth* channel: visual salience—chromatic vividness, compositional legibility, and recognizable subject matter—widens the set of collectors who can form a private value for the lot. Because an ascending auction converts the number of active bidders into both the probability of clearing the reserve price and the height of the final bid, works whose appearance appeals to a broader audience should exhibit lower failure rates and stronger upside relative to the estimate. The second is a *canonicity* channel: within a given artist's oeuvre, buyers pay a premium for works that are stylistically representative of the period for which that artist is celebrated, and discount transitional, derivative, or atypical works. Style typicality is a visual property that hedonic dummies for artist, medium, and year cannot express, yet it is precisely what distinguishes a signature canvas from an outlier by the same hand in the same year. The third is an *information-asymmetry* channel: condition, surface handling, and execution quality are legible to specialists who inspect the work but are absent from tabular catalog variables, so they survive in the hedonic residual and appear to the econometrician as unexplained valuation deviation. Under all three channels, visual features should predict not only price levels—the outcome studied by most prior work—but also the two risk quantities of interest here, namely the probability of transaction failure and the dispersion of realized price around the expert estimate. Section 3.2 formalizes these channels, and the ablation and interpretability analyses in Sections 6.4 and 6.6 test them empirically.

Against this backdrop, this paper proposes a multimodal deep learning framework that integrates painting image features, auction transaction data, artist information, and market variables, and develops a valuation deviation regression model and an unsold-lot classification model to quantify price-deviation risk and transaction-failure risk, respectively, for art financial assets. The primary contribution of this paper lies in incorporating the visual heterogeneity of non-standard assets into financial risk assessment, thereby providing a more actionable quantitative foundation for artwork-collateralized financing, wealth management allocation, and auction pricing strategy.

## 2 Literature review

### 2.1 Art asset pricing and financial risk

Artwork has entered the purview of financial research primarily because it possesses the compound attributes of aesthetic consumption, wealth storage, and investment allocation. Unlike standardized assets such as stocks and bonds, artworks do not generate stable cash flows, and their value derives primarily from scarcity, artist reputation, cultural capital, and collector preferences. Campbell (2008) earlier defined art as an alternative asset class, noting its low correlation with traditional financial assets and a degree of diversification value. Goetzmann et al. (2011) further found that changes in stock market returns and top-income levels affect art prices through a long-term art index, suggesting that the art market is not independent of the macro-financial environment but is closely related to wealth creation, income distribution, and risk appetite. Reports from Art Basel and UBS confirm that art remains a significant allocation target in high-net-worth (HNW) wealth management, and that despite cyclical fluctuations in sales volume, trading activity has demonstrated a degree of resilience (McAndrew, 2025a).

The risk structure of art as a financial asset, however, differs substantially from that of conventional financial instruments. First, art transactions are infrequent, and many works have no public trading record for long periods, which makes price series discontinuous. Second, public databases mainly record traded samples, while unsold works and private transactions are difficult to observe; this asymmetric observability introduces selection bias into return estimates. Korteweg et al. (2016) point out that relying only on repeat-transaction samples may distort estimated returns on art investment. In addition, returns on art are not purely financial; they also encompass aesthetic enjoyment, social-status signaling, and the intrinsic utility derived from collecting. Renneboog and Spaenjers (2013) describe this non-financial utility as “the purchase of beauty,” implying that investors may accept a risk-return trade-off that differs from that of conventional financial assets.

From a risk-management perspective, the principal challenges of art financialization stem from valuation uncertainty and constrained liquidity. The transaction model developed by Lovo and Spaenjers (2018) shows that low liquidity may result from sellers' timing decisions, difficulties in matching buyers, and other market frictions. Beggs and Graddy (2008) examine anchoring and buy-in phenomena in auctions: when the highest bid is below the reserve price, the work may be withdrawn or bought in by the auctioneer, leading to a failed transaction. Such cases suggest not only a mismatch between valuation and demand, but also weaker market sentiment. Risk assessment must therefore extend beyond the final sale price to consider whether the realized price falls outside the estimated valuation range and whether the asset can actually be transacted.

### 2.2 Traditional non-standard asset valuation methods: applications and limitations of the hedonic model and the repeat-sales model

Traditional art valuation often relies on hedonic pricing models and repeat-sales methods. Under the hedonic framework, artworks are treated as combinations of observable attributes such as artist, size, medium, subject matter, date of creation, auction house, and sale location, and regression analysis is used to estimate the marginal effect of each attribute on price. Chanel et al. (1996) were among the first to apply hedonic price indices to the painting market. Collins et al. (2009) further show that variables such as subject matter, size, and medium can be important predictors of art prices. This approach therefore offers strong explanatory power and is well-suited for constructing price indices and identifying price determinants.

Nevertheless, hedonic pricing models have clear limitations. First, they often assume a linear or additive relationship between attributes and price, which makes it difficult to capture interactions among artist reputation, work quality, market cycles, and aesthetic preferences. Second, many important visual details cannot be fully represented by manually defined variables. Two oil paintings of similar size and period may differ sharply in price because of differences in composition, color, brushwork, and stylistic maturity. Ma et al. (2022) find that color composition can influence the auction value of paintings through emotional-pleasure channels, suggesting that visual elements are not merely aesthetic attributes but can also function as sources of financial premiums.

The repeat-sales method focuses on price changes for the same artwork sold at different points in time, thereby helping to control for quality differences. Mei and Moses (2002) construct an art price index based on this approach, which has contributed to research on art investment returns. However, this method requires the same artwork to appear in public sales multiple times, so the available sample is usually small and tends to over-represent works with higher visibility and greater liquidity. Wang (2018) compares hedonic pricing, repeat-sales, and hybrid models in the Chinese painting market, showing that different methods may produce different estimates of returns and volatility, which indicates that traditional valuation estimates are sensitive to model specification and sample composition. Galbraith and Hodgson (2018) combine hedonic pricing with repeat-sales information, further showing that a single method often struggles to balance predictive accuracy, sample coverage, and economic interpretation.

### 2.3 Applications of AI and deep learning in financial risk early warning

Compared with research on art finance, the application of artificial intelligence to risk early warning in standard financial markets is considerably more mature. Machine learning can identify complex patterns in high-dimensional, non-linear, and non-stationary data, and is therefore widely used in stock price forecasting, credit scoring, default identification, fraud detection, and portfolio optimization. Kou et al. (2014) discuss the evaluation of clustering algorithms in financial risk analysis, demonstrating that machine learning can not only predict outcomes but also support the clustering of risk objects. Krauss et al. (2017) applied deep neural networks, gradient-boosted trees, and random forests to statistical arbitrage of S&P 500 constituents, finding that non-linear models can extract effective signals from large-scale financial data. A comprehensive review by Ozbayoglu et al. (2020) further demonstrates that deep learning has expanded into multiple financial domains, including algorithmic trading, credit risk assessment, sentiment analysis, and fraud detection. Recent transaction-security studies further show that temporal attention, spatial–temporal gating, hierarchical gated networks, behavioral representation learning, and GAN-based hybrid sampling can improve the modeling of non-linear financial-risk patterns in transactional data (Xie et al., 2023). More recently, deep learning approaches have continued to advance across diverse financial risk domains. Chen and Yang (2026) propose a real-time dynamic graph learning framework with temporal attention for financial fraud detection, reporting substantial improvements in detection accuracy over industry-standard baselines. Al-Besher and Al-Najjar (2026) show that neural networks outperform traditional GARCH models in capturing non-linear volatility patterns in stock markets. Raliphada et al. (2026) conduct a systematic review of transformer-based NLP approaches for credit risk prediction, while Jehnen et al. (2026) introduce a domain-specific sentence-transformer for extracting predictive latent topics from financial disclosures. Alnajjar et al. (2026) further demonstrate the utility of combining econometric and machine learning methods for forecasting banking stability.

The advantage of deep learning over traditional methods lies in its ability to learn features automatically. Convolutional neural networks are suitable for extracting spatial features from images, recurrent neural networks and long short-term memory networks are suitable for processing time series, and attention mechanisms and Transformers can learn dynamic weights between variables. Mienye and Jere (2024) note that deep learning still faces several challenges in financial applications, including limited interpretability, overfitting risk, and data-quality concerns. Even so, it has strong potential for processing multi-source heterogeneous information and non-linear relationships. For artworks, which constitute a prototypical non-standard asset class, deep learning holds the potential to improve price-prediction accuracy and to transform visual, market, and transaction information into quantifiable risk signals that are difficult to encode explicitly through conventional methods.

### 2.4 Research gap

Previous studies in art finance have established a solid foundation in return measurement, index construction, and auction mechanism analysis. However, the use of image-level features remains limited. Most studies rely mainly on structured variables such as artist, dimensions, medium, auction house, and sale date. Even when subject matter or artistic movement is included, these variables usually come from manual labels, making it difficult to capture the visual details of artworks. Aubry et al. (2023) train neural networks by combining visual and non-visual features; their use of machine-predicted prices to identify valuation biases in auction houses demonstrates that image-level information can be meaningfully incorporated into financial research. Indrawan et al. (2023) also propose a method that combines painting image features with transaction table data. More recent work by Mei et al. (2025) further shows that deep visual embeddings can contribute to art market valuation.

Based on the foregoing review, three principal gaps can be identified in the existing literature. First, many studies use machine learning mainly to predict final sale prices, while few incorporate both valuation deviations and unsold lots into a unified risk framework. Second, liquidity research has largely remained at the level of market-wide institutions, and relatively few studies treat the probability of unsold lots as an individual-level, predictable risk. Third, the interplay among computer vision features, structured financial variables, and auction mechanisms has not been sufficiently examined. Mauer and Paszkiel (2024) compare different tabular-data models for predicting art auction results; their findings suggest that while traditional machine learning models serve as useful benchmarks, tabular variables alone are insufficient to explain how visual aesthetics translate into market risk. This paper therefore constructs a multimodal deep learning framework by integrating artwork images, artist information, auction transaction variables, and macroeconomic conditions. Valuation deviation regression and unsold-lot classification are then employed to characterize the dual financial risks inherent in art assets.

## 3 Theoretical framework

### 3.1 Defining risk dimensions of art financial assets

The identification of risks associated with art financial assets must be grounded in both market mechanisms and the inherent characteristics of the assets themselves. Artworks differ from securities because securities are usually supported by continuous pricing, whereas the value discovery of artworks mainly takes place through auctions, galleries, and private transactions. Among these channels, the auction market is most often used as the primary data source for empirical research because its records are relatively transparent. Even so, auction prices should not be regarded as frictionless market values; they may be affected by valuation ranges, reserve prices, the number of bidders, auction house reputation, and broader market sentiment. On this basis, the risks of art financial assets can be divided into valuation risk and liquidity risk. These two dimensions are closely connected within the same auction mechanism: valuation risk reflects whether the price assessment is accurate, while liquidity risk reflects whether the work can actually be traded. As summarized in Table 1, prior studies provide the theoretical basis for the identification of valuation risk, liquidity risk, and comprehensive financial risk in this study.

**Table 1:** Summary of related literature and implications for this paper.

Research topic	Representative literature	Key contributions	Implications for this paper
Art as an Alternative Asset and Returns	Campbell (2008); Goetzmann et al. (2011); Korteweg et al. (2016)	Discusses the value of art portfolio allocation, the wealth effect, and biases in return estimates	Demonstrates that art can be financialized, but requires addressing low-frequency trading and sample selection
Traditional Valuation and Index Methods	Chanel et al. (1996); Mei and Moses (2002); Wang (2018)	Establishes models for characteristic prices, repeat transactions, and composite indices	Provides benchmark methods while highlighting the limitations of linear settings and insufficient sample coverage
Auction Mechanisms and Liquidity	Beggs and Graddy (2008); Lovo and Spaenjers (2018)	Explains anchoring, buy/no-sale outcomes, and transaction frictions	Supports the use of the no-sale rate as a proxy for art liquidity risk
Financial AI and Risk Warning	Kou et al. (2014); Krauss et al. (2017); Ozbayoglu et al. (2020); Xie et al. (2023); Chen and Yang (2026); Al-Besher and Al-Najjar (2026); Raliphada et al. (2026); Jehnen et al. (2026); Alnajjar et al. (2026)	Demonstrates the application of machine learning and deep learning in financial forecasting	Provides methodological insights into modeling risks in non-linear, multi-source data
Multimodal Artwork Valuation	Aubry et al. (2023); Indrawan et al. (2023); Mei et al. (2025)	Incorporates visual features into art price forecasting and the identification of valuation biases	Forms the direct foundation for this study's interdisciplinary approach spanning finance and computer vision

Valuation risk refers to the gap between the actual hammer price and the lower bound of the auction house estimate. The lower bound is adopted as the benchmark because auction houses typically provide an estimated price range, and this lower limit reflects the minimum value deemed acceptable by expert appraisers; it is also close to the reserve price and the seller's expected price. When the final price falls below this lower bound, or remains very close to it, market recognition is weaker than suggested by the pre-auction valuation, and investors may face asset-revaluation risk and a decline in collateral value. Aubry et al. (2023) demonstrate that auction house estimates are not entirely objective, and that a systematic relationship exists between machine-predicted prices and auction outcomes. Using the degree of deviation of the final sale price from the lower limit of the estimate as a valuation-risk variable is therefore consistent with the auction system and allows expert valuation errors to be converted into a modelable indicator.

The term “liquidity risk” as used in this paper refers to the risk that a work of art cannot be sold at a reasonable price at a specific auction time. Because the art market lacks continuous bid-ask quotes, traditional financial liquidity metrics such as bid-ask spreads, turnover rates, or instantaneous transaction costs are difficult to apply directly. “Unsold” or “buy-back” outcomes in auctions provide observable proxy variables: when the highest bid fails to meet the reserve price and the work remains unsold, it implies that the seller is unable to realize value at that point in time. Beggs and Graddy (2008) note that anchoring, reserve prices, and buy-ins influence auction outcomes, and Lovo and Spaenjers (2018) explain that low liquidity in the art market stems from matching frictions, timing of sales, and heterogeneous buyer preferences. This paper therefore uses the unsold rate, or the probability that a work goes unsold, as a proxy for liquidity risk, which possesses clear economic significance and data feasibility.

Three properties justify this proxy on economic grounds. First, it is *incentive-compatible with a genuine liquidation attempt*. Consigning a work to a major evening or day sale is costly and irreversible in reputational terms: the seller pays cataloging and marketing costs, accepts a binding reserve, and exposes the work to a public record of failure that depresses its future marketability—the “burned” phenomenon. A consignment therefore reveals a sincere intention to convert the asset into cash at the prevailing market price. When such an attempt fails, the failure is informative about the asset's tradability rather than about the seller's indifference. Second, the outcome is *measured at the individual-asset level and at a known date*, which is precisely the unit at which a collateral or portfolio decision is taken. A lender does not need to know the average turnover of the Post-War and Contemporary category; it needs to know whether this canvas, pledged against this loan, would clear at a sale held this season. Third, failure is *economically consequential, not merely statistical*. An unsold lot interrupts price discovery, leaves the asset without a refreshed mark, typically imposes a cooling-off period of one to several years before reconsignment is advisable, and—because the failure is publicly observable—shifts the distribution of prices attainable in any subsequent attempt downward. Each of these consequences maps directly onto the horizon over which a secured lender expects to recover principal.

The proxy also differs from conventional liquidity measures in ways that are worth making explicit, since the difference is one of kind rather than of precision. Standard microstructure measures characterize the *cost* of executing a trade conditional on execution being feasible: the bid-ask spread prices immediacy, Amihud illiquidity prices the return impact per unit of volume, and turnover measures how frequently a float changes hands. All three presuppose a continuously quoted, fungible instrument with many identical units. Artworks satisfy none of these preconditions: each lot is unique, so there is no float to turn over; there are no standing two-sided quotes, so no spread exists between auctions; and volume is not defined for a single object. What the art market offers instead is a discrete, scheduled liquidity event with a publicly observed binary outcome. The unsold indicator therefore measures the *feasibility* of execution rather than its cost—closer in spirit to the fill probability of a limit order resting at the reserve price, or to the time-to-sale hazard used in real-estate and private-asset research, than to a spread. This distinction has a practical consequence for interpretation: a rising unsold probability should not be read as an increase in transaction costs that a patient seller can avoid by trading more slowly, but as a decline in the probability that the asset can be monetized at all within the seller's intended window. For a secured lender, this is the more binding constraint, because it governs whether the collateral can be liquidated during the workout period rather than merely how much of the proceeds are lost to friction. A secondary and complementary measure—the deviation of the realized price from the low estimate, defined above as the valuation-risk variable—then captures the price concession required conditional on a sale occurring. Taken together, the two variables recover the two dimensions that spread-based measures bundle into a single number for standardized assets: whether a trade happens, and at what discount. The three risk dimensions, their corresponding proxy variables, economic implications, and expected model tasks are summarized in Table 2.

**Table 2:** Risk dimensions of art financial assets and corresponding model tasks.

Risk dimension	Operational definition	Proxy variable	Economic implication	Expected model task
Valuation risk	The extent to which the final sale price deviates from the lower limit of the auction house's estimate	Final sale price/lower limit of estimate; price deviation rate	Reflects the gap between expert valuations, market demand, and actual willingness to pay	Regression forecast
Liquidity risk	The probability that a lot will fail to sell at a reasonable price at the time of auction	Whether a lot goes unsold, the unsold rate, and the buy-in status	Reflects failed transactions, difficulties in realizing value, and disruptions in price discovery	Binary classification prediction
Comprehensive financial risk	Risks arising from valuation deviations and failed trades	Risk scores or dual-output results	Supports collateralized financing, asset allocation, and auction strategies	Dual-model or multi-task learning

### 3.2 Deep learning-driven feature fusion mechanism

The value of artworks is determined not by a single variable but by the combined effects of visual quality, artist reputation, the auction system, and macroeconomic conditions. Traditional models typically use structured variables such as the artist's name, dimensions, medium, year of creation, auction house, and time of sale, which can describe the external attributes of a work but struggle to capture its internal visual structure. Compositional balance, color saturation, brushstroke density, subject placement, and stylistic consistency within a painting's imagery often directly influence collectors' aesthetic judgments and willingness to pay. Ma et al. (2022) demonstrate, through their research on color and emotion, that visual elements can influence auction value through emotional channels; image features should therefore be regarded as core information in financial risk assessment.

It is useful to state the mechanism more precisely, because it determines what the visual branch is expected to add over and above a well-specified hedonic model. Let a lot be described by observable catalog attributes *x*_*i*_ and by a latent quality vector *q*_*i*_ that is legible in the image but absent from the catalog. A conventional hedonic regression estimates 𝔼 [log*P*_*i*_ ∣ *x*_*i*_] and consigns *q*_*i*_ to the error term. The three channels identified in the Introduction correspond to three distinct ways in which *q*_*i*_ enters the outcome. Under the demand-breadth channel, *q*_*i*_ shifts the number of bidders *n*_*i*_ who draw a private value above the reserve; since in an ascending auction the expected hammer price is the second-highest of *n*_*i*_ order statistics and the probability of clearing the reserve is increasing in *n*_*i*_, visual salience raises the conditional mean of *r*_*i*_ and lowers $\mathrm{Pr}\left(y_{i}^{L}=1\right)$ simultaneously—which is why a single shared representation feeding two heads is the natural specification rather than two unrelated models. Under the canonicity channel, *q*_*i*_ interacts with artist identity: the premium for stylistic typicality is defined only relative to the body of work for which a given artist is known, so its effect is an interaction between a visual embedding and an artist fixed effect, a term that is not estimable in a linear model with the sample sizes available per artist but that a fusion layer can approximate. Under the information-asymmetry channel, *q*_*i*_ is observed by bidders inspecting the work but not by the analyst, so it generates precisely the residual dispersion that the valuation-deviation task is designed to measure; recovering even part of *q*_*i*_ from the image should therefore reduce the conditional variance of *r*_*i*_, which is what the RMSE comparison in Section 6.3 tests. This decomposition also clarifies the null hypothesis under which visual features would add nothing: it holds only if catalog attributes are sufficient statistics for *q*_*i*_, that is, if artist, medium, size, and date already exhaust everything a bidder learns by looking at the work. The ablation results in Section 6.4 reject this restriction, and the attribution analysis in Section 6.6 identifies which visual regularities the model actually exploits.

Deep learning offers a promising avenue for addressing these limitations. Convolutional neural networks or visual Transformers can be used to extract high-dimensional visual embeddings from painting images, capturing style, texture, and composition features that are difficult to describe through manual labels. At the same time, structured models can process tabular variables such as artist reputation, artwork dimensions, medium, estimated price range, auction house, auction year, and macroeconomic indicators. The key to a multimodal framework is to place these two types of features into a shared representational space, allowing the model to connect how an artwork looks with how the market prices it. Indrawan et al. (2023) show that combining image features with transaction data can support painting price prediction, and from a financial-empirical perspective Aubry et al. (2023) further demonstrate that joint visual-non-visual models can reveal auction house estimation biases in the market.

The proposed theoretical framework consists of three layers, as shown in Figure 1. The first layer is the information-input layer, which includes image information, artist and artwork information, auction transaction information, and external market information, providing a more complete view of the available data. The second layer is the feature-fusion layer: deep visual models extract image embeddings while structured models process tabular variables, and through this layer the model learns the interactive relationships between visual and non-visual information that are expected to be important for final prediction. The third layer is the risk-output layer, which contains two tasks: valuation regression and unsold classification. The valuation regression task predicts how much the final sale price deviates from the lower bound of the estimated price range, while the unsold classification task predicts whether the work may remain unsold. These two tasks are not independent: overvaluation may increase the probability of a work going unsold, while a history of unsold outcomes may affect future valuation expectations. Both tasks are therefore integrated into a unified framework to evaluate art as a financial asset from the dual perspectives of price risk and transaction risk.

**Figure 1:**
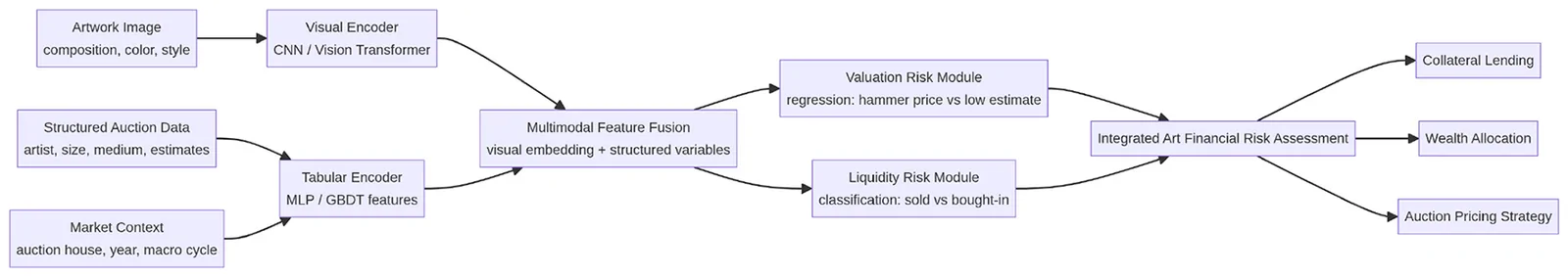
Multimodal deep learning-driven risk assessment framework for art financial assets.

This framework carries theoretical significance in that it translates the aesthetic heterogeneity of artworks into quantifiable financial risk signals. For financial institutions, the model can support the estimation of collateral haircuts; for auction houses, it can help calibrate valuation ranges and provide early warnings for lots with higher unsold risk; and wealth managers may use it to identify assets with high valuation uncertainty and low liquidity. In this framework, deep learning is not treated as a purely technical black box. Instead, it is embedded within a structure that combines non-standard asset pricing, auction liquidity, and multi-source information fusion, offering an interdisciplinary approach to art finance research that links financial theory with computational methods.

## 4 Data and feature engineering

### 4.1 Data sources and sample construction

The data design in this paper is structured around three levels—“auction transactions,” “artwork images,” and “macro-financial environment”—to ensure that the valuation and liquidity risks of art as a financial asset can be analyzed within a single framework. Transaction-level data were sourced from publicly disclosed auction records for modern and contemporary paintings, works on paper, and mixed-media works offered by Sotheby's, Christie's, and Phillips, covering the 24-year interval from January 2000 to December 2023. Auction data were selected as the primary sample because the auction market provides traceable information such as public estimate ranges, hammer prices, unsold status, and auction dates, making it suitable for empirical financial research. Renneboog and Spaenjers (2013) analyze the determinants of art prices based on large-scale auction transactions, demonstrating that auction records can provide a relatively standardized data foundation for measuring art returns and risks; Oosterlinck (2017) also treats artworks as investable assets, emphasizing the importance of publicly available transaction data in assessing the asset attributes of artworks.

The sample is constructed using auction lots as the observation unit, recording variables such as artwork title, artist, low estimate, high estimate, hammer price, unsold status, auction house, auction location, auction date, year of creation, dimensions, medium, subject matter, and image links. For sold samples, the hammer price is uniformly converted to U.S. dollars at the exchange rate prevailing on the sale date and deflated to constant 2023 dollars using the U.S. CPI; for unsold samples, the estimate range, artwork attributes, and image information are retained to construct liquidity-risk classification labels. Retaining unsold lots is essential to the design: because these lots are precisely the observations discarded by repeat-sales and most hedonic studies, a sample restricted to completed transactions cannot identify transaction-failure risk at all (Korteweg et al., 2016).

Table 3 reports the construction of the estimation sample. The initial extraction yielded 214,806 lot records. Records were then excluded for the following reasons, applied in the order listed: no catalog image available or the image resolution fell below 224 × 224 pixels (61,143 lots); missing or unparseable dimensions, medium, or execution date (28,457); absent published estimate range, which removes lots offered without estimate and most single-owner charity sales (14,902); prices denominated in currencies without a reliable daily reference rate for the sale date (5,218); and lots whose low estimate fell outside the 0.5th–99.5th percentile range of the pooled estimate distribution, a symmetric trim that removes both nominal lots and a small number of exceptional masterpieces whose pricing dynamics are not representative (8,772). The final analysis sample comprises 96,314 lots by 11,268 distinct artists, of which 69,572 sold (72.2%) and 26,742 went unsold or were bought in (27.8%). This unsold share is consistent with the aggregate buy-in rates reported for the modern and contemporary segment over the sample period and confirms that transaction failure is a first-order rather than a marginal phenomenon; the classification task is therefore moderately imbalanced but does not require the extreme rare-event treatment used in fraud detection.

**Table 3:** Sample construction and partition.

Step/partition	Lots	Share
Panel A: Sample construction		
Initial extraction (Sotheby's, Christie's, Phillips; 2000–2023)	214,806	100.0%
Less: image unavailable or below 224 × 224 pixels	−61,143	28.5%
Less: missing dimensions, medium, or execution date	−28,457	13.2%
Less: no published estimate range	−14,902	6.9%
Less: non-convertible currency on sale date	−5,218	2.4%
Less: low estimate outside 0.5th–99.5th percentile	−8,772	4.1%
Final analysis sample	96,314	44.8%
Sold	69,572	72.2%
Unsold or bought in	26,742	27.8%
Panel B: Chronological partition		
Training set (2000–2018)	67,419	70.0%
of which unsold	18,514	27.5%
Validation set (2019–2020)	11,286	11.7%
of which unsold	3,262	28.9%
Test set (2021–2023)	17,609	18.3%
of which unsold	4,966	28.2%

Because the exclusion criteria are not random with respect to the outcome, their consequences for inference are worth stating. The image-availability filter is the largest single restriction and binds most heavily on early-period day sales and on lower-value lots, for which houses historically digitized fewer catalogs. The resulting sample is thus tilted toward better-documented and higher-value material, which likely renders the reported unsold rate a conservative estimate of the true failure rate in the full population, since sparsely documented lots by thinly traded artists fail more often. Section 6.7 addresses this by re-estimating the model separately on the lower-liquidity artist group and on estimate quartiles, and Section 7.5 discusses the residual selection concern that no within-sample correction can fully resolve.

Model selection follows a strictly chronological partition rather than a random split, because a random split would allow the model to learn from auction outcomes that postdate the observations it is asked to predict, and would also permit near-duplicate works by the same artist consigned in the same season to appear on both sides of the partition. Lots offered between January 2000 and December 2018 constitute the training set (67,419 lots), those from January 2019 to December 2020 the validation set used for hyperparameter selection and early stopping (11,286 lots), and those from January 2021 to December 2023 the held-out test set on which all reported results are computed (17,609 lots). The test window is deliberately retained despite spanning the pandemic-era market disruption and the subsequent 2022–2023 correction, since a framework intended for risk management should be evaluated under exactly such conditions rather than only in tranquil markets. All artist-level history variables are constructed from a rolling window that ends strictly before each lot's sale date, so no observation is informed by its own future or by the future of its artist. Where an artist appears in more than one partition, only the artist's pre-partition transaction history enters the features, which preserves the temporal ordering while retaining the reputation information that is central to art pricing.

The two tasks are estimated on different subsets of this partition, a distinction that matters for interpreting the reported metrics. Liquidity-risk classification uses all 96,314 lots, since the unsold indicator is defined for every observation. Valuation-deviation regression is necessarily restricted to the 69,572 sold lots, because the dependent variable *r*_*i*_ requires a realized hammer price; the corresponding training, validation, and test counts are 48,905, 8,024, and 12,643. This restriction is itself a selection mechanism—the regression is estimated on lots that cleared their reserve—and the joint framework partially addresses it, since the classification head models the selection event that determines regression-sample membership.

Macro-financial data are incorporated to characterize external market conditions prevailing at the time of each auction. This paper matches the S&P 500 index return, risk-free interest rate, inflation rate, VIX fear index, consumer confidence index, and market liquidity indicators for the month of the auction. Since art prices are influenced by the wealth effect and the confidence of high-income groups, Renneboog and Spaenjers (2013) find that high-income consumer confidence and art market sentiment can predict trends in art prices, and Penasse et al. (2014) further emphasize the role of sentiment factors in art price formation. Bouri et al. (2018) indicate that global fear indices may influence the art market; incorporating variables such as the VIX into feature engineering therefore helps capture changes in macro-level risk appetite.

### 4.2 Multimodal feature extraction scheme

The core features in this paper comprise visual features, transaction features, and macro-financial features. Visual features constitute unstructured data extracted primarily from auction catalog images. Specifically, the images are first uniformly downloaded, deduplicated, and quality-screened, then resized to the input dimensions required by the pre-trained model and normalized. This paper uses ResNet-50, initialized from ImageNet pre-trained weights, as the backbone network; after removing the final classification layer, the 2048-dimensional output of the global average pooling layer is taken as the deep visual feature vector. ResNet alleviates the training-degradation problem in deep networks through residual connections and is a classic model in image recognition (He et al., 2016). Importantly, the backbone is not used as a frozen ImageNet extractor: its later residual stages are subsequently fine-tuned on the auction sample so that the representation adapts to the art domain, as detailed in Section 5.4. As a robust alternative, VGG16 is also used to extract visual embeddings; Simonyan and Zisserman (2015) demonstrate the transferability of representations from deep convolutional networks. Within the art image domain, Cetinic et al. (2018) show that fine-tuned convolutional neural networks can be used for art classification, providing a methodological basis for this paper's use of pre-trained CNNs to extract visual features from paintings.

Transaction features are organized into three categories: artist-level, artwork-level, and auction-level variables. Artist-level variables characterize historical reputation and market recognition, such as the average transaction price over the past 3 years, the number of transactions, historical unsold rates, and price growth rates. Artwork-level variables include area, aspect ratio, year of creation, age of the work, material, medium, and subject matter. Auction-level variables include the auction house, auction location, auction season, width of the estimate range, and median estimate. Macro-financial features are matched by auction month to reflect the wealth effect, cost of capital, and market risk appetite. Finally, multimodal features are standardized or encoded before being fed into the model: continuous variables are standardized using z-scores, categorical variables are encoded using one-hot encoding or embedding, and image vectors are input into the fusion network as high-dimensional visual representations.

## 5 Methodology

### 5.1 Multimodal neural network

This paper employs a multimodal neural network to process artwork images and structured financial data simultaneously. The fundamental logic behind this design is as follows: the image branch is responsible for extracting visual information from the artwork itself, while the structured branch extracts information regarding the artwork's attributes, auction conditions, and macroeconomic environment; the fusion layer, in turn, learns the interactive relationships between these two types of information. LeCun et al. (2015) note that deep learning can automatically discover complex structures from raw data through multi-layer representation learning, and Baltrušaitis et al. (2019) summarize multimodal learning as the representation, alignment, and fusion of different information sources. Art financial assets are characterized by multi-source heterogeneity, making them well-suited for multimodal fusion networks rather than simple linear regression or single-tree models. This treatment is also consistent with transaction-fraud detection research, in which binary risk labels are modeled through behavioral representation learning and gated deep networks (Xie et al., 2023).

The image branch uses a pre-trained ResNet-50 as its backbone. Let the image of the *i*-th artwork be *I*_*i*_; the CNN encoder produces a visual vector *v*_*i*_ = *f*_cnn_(*I*_*i*_). In the art market, where sample sizes are limited, training deep networks from scratch is prone to overfitting. This paper therefore adopts a partial transfer-learning strategy: the parameters of the front convolutional layers are frozen and only the rear residual blocks are fine-tuned, so that the general edge, texture, and shape features learned from large-scale image data are preserved while the representation retains the capacity to adapt to stylistic variations in art images. The precise staging of this procedure, and a comparison against both a fully frozen and a fully unfrozen backbone, are reported in Sections 5.4 and 6.5. For robustness testing with VGG16, the activation vectors before its fully connected layers are used as an alternative visual representation.

The structured branch uses a multi-layer perceptron to process transaction variables and macroeconomic variables. Let the structured features be *x*_*i*_, which include the artist's historical performance, artwork dimensions, materials, year of creation, auction house, estimate range, and macroeconomic indicators. Categorical variables are first converted into numerical vectors via one-hot encoding or an embedding layer, while continuous variables are standardized before being fed into a fully connected network to obtain the structured representation *s*_*i*_ = *f*_mlp_(*x*_*i*_). Kraus and Feuerriegel (2017) demonstrate that deep neural networks and transfer learning can be used for financial decision support, indicating that MLPs can play a role in processing structured financial information.

The fusion layer employs a post-fusion approach, concatenating the visual and structured vectors into *z*_*i*_ = [*v*_*i*_; *s*_*i*_], which is then fed into several fully connected layers to generate a shared representation *h*_*i*_. The advantage of this design is that it preserves the independent encoding processes for both types of information while allowing the model to learn the non-linear interaction between visual quality and market conditions at higher levels. For example, a work with strong visual impact is likely to carry lower valuation risk only when the artist's reputation, the valuation range, and market risk appetite all align; conversely, even a work of high visual quality may fail to sell if the valuation is too high or market sentiment weakens.

### 5.2 Task 1: liquidity risk prediction model

Liquidity-risk prediction is formulated as a binary classification task, with the objective of determining whether a particular artwork will go unsold at auction. Let $y_{i}^{L}=1$ denote that the artwork goes unsold or is bought in, and $y_{i}^{L}=0$ denote that the artwork is successfully sold. The model takes the shared representation *h*_*i*_ as input and passes it through a sigmoid output layer to obtain the probability of an unsold outcome, *p*_*i*_ = σ(*W*_*L*_*h*_*i*_ + *b*_*L*_). This probability can be interpreted as the conditional probability that the artwork will not be sold, given the specified image, transaction attributes, and macroeconomic environment.

The loss function employs binary cross-entropy:


$$\begin{array}{c} L_{L}=-\frac{1}{N}\overset{N}{\underset{i=1}{\sum }}\left[y_{i}^{L}\log p_{i}+\left(1-y_{i}^{L}\right)\log\left(1-p_{i}\right)\right] \end{array}$$ (1)

Binary cross-entropy is the standard objective function for binary classification neural networks, as it penalizes deviations between predicted probabilities and true labels (Goodfellow et al., 2016). In financial risk research, tasks such as default identification, fraud detection, and credit scoring are typically formulated as binary classification problems. Lessmann et al. (2015) conduct a systematic comparison of credit-scoring classification algorithms, demonstrating that classification models have mature applications in financial risk early warning. This paper transforms the risk of unsold lots into a binary classification task analogous to default probability; this is not a simple application of existing techniques, but is grounded in the economic implication that artworks “cannot be liquidated at a reasonable price.”

### 5.3 Task 2: valuation deviation risk prediction model

Valuation deviation risk is formulated as a regression task, with the objective of predicting the degree of deviation between the final sale price and the lower limit of the auction house's estimate. Let the final sale price be *P*_*i*_ and the lower limit of the estimate be $E_{i}^{\text{low}}$; the valuation deviation variable is then defined as shown in Equation 2:


$$\begin{array}{c} r_{i}=\log P_{i}-\log E_{i}^{\text{low}} \end{array}$$ (2)

When *r*_*i*_ < 0, the transaction price is below the lower valuation threshold, suggesting a risk of downward valuation for the artwork; when *r*_*i*_ is close to zero, the transaction price is close to the experts' lowest valuation; and when *r*_*i*_ > 0, the market's willingness to pay exceeds the lower valuation threshold. The advantage of using logarithmic differences is that they mitigate the right-skewed nature of the price distribution and place the degree of deviation for artworks at different price levels on a relatively comparable scale.

After passing through the shared representation layer *h*_*i*_, the model feeds into a linear output layer to obtain the predicted valuation deviation ${\hat{r}}_{i}=W_{R}h_{i}+b_{R}$ as defined in Equation 3. The loss function uses mean squared error:


$$\begin{array}{c} L_{R}=\frac{1}{N}\overset{N}{\underset{i=1}{\sum }}{\left({\hat{r}}_{i}-r_{i}\right)}^{2} \end{array}$$ (3)

MSE is appropriate for regression tasks involving continuous variables and imposes a higher penalty on large deviations (Goodfellow et al., 2016). Given the fat-tailed distribution and extreme high prices observed in art prices, robustness tests can employ the Huber loss or apply quantile truncation to high-priced samples. Bouri et al. (2018) note that global panic variables are predictive of the art market, suggesting that valuation deviations may be non-linearly influenced by macroeconomic risk appetite; multimodal deep networks are better suited than linear models for capturing such complex relationships.

This paper employs two training strategies. The first is a dual-model strategy, in which a failed-auction classification network and a valuation regression network are trained separately to clearly compare the predictive capabilities of each task. The second is a multi-task learning strategy, in which the image branch, structured branch, and fusion layer are shared, with classification and regression heads placed at the top. The total loss function can be written as:


$$\begin{array}{c} L=\lambda L_{L}+\left(1-\lambda \right)L_{R} \end{array}$$ (4)

Here λ is a weighting parameter used to balance liquidity risk and valuation risk. The advantage of multi-task learning lies in the fact that the two risk dimensions may share the same set of market signals; for example, overvaluation may result in a transaction price below the lower bound of the valuation range, as well as an increased probability of a lot going unsold. Aubry et al. (2023) have demonstrated that combining visual and non-visual features can identify valuation biases in auction houses. This paper extends that approach into a dual-risk output framework, enabling the model not only to predict prices but also to identify the risk of transaction failure. The use of generative and sampling-based mechanisms in fraud-risk research further suggests that data imbalance and rare-event modeling can be addressed through hybrid sampling and time-aware generative networks.

Because the regression loss is defined only for sold lots, the two terms in Equation 4 are evaluated on overlapping but unequal subsets of each mini-batch. The classification term is computed over all lots in the batch, whereas the regression term is masked to the sold subset and normalized by the number of sold lots in that batch rather than by the batch size, so that the effective weight on the regression objective does not fluctuate with the realized sold fraction. This masking is what allows unsold lots to inform the shared representation—they contribute gradient signal through the classification head and thereby shape the encoder used by the regression head—without contaminating the regression loss with undefined targets.

### 5.4 Implementation details and training protocol

The model was implemented in PyTorch 2.1 (open-source software maintained by Meta AI, Menlo Park, CA, United States) and trained on a single NVIDIA A100 (40 GB) GPU. This subsection documents the configuration in sufficient detail to permit replication; Table 4 summarizes the settings, and the discussion below records the reasoning behind the choices that are not conventional defaults.

**Table 4:** Training configuration and hyperparameter search space.

Component	Setting	Search space/note
Framework and hardware	PyTorch 2.1; 1 × NVIDIA A100 40 GB	—
Visual backbone	ResNet-50, ImageNet-1k initialization	VGG16 tested as alternative
Backbone adaptation	Stage 1: fully frozen, 10 epochs. Stage 2: layer3–layer4 unfrozen, 30 epochs	Frozen-only variant reported in Table 8
Image input	Resize short edge 256, center crop 224 × 224, ImageNet normalization	–
Augmentation	Horizontal flip; random resized crop [0.9, 1.0]	Color jitter and rotation excluded by design
Visual projection	2048 → 256 (BN + ReLU)	–
Structured branch	187 → 256 → 128	Artist embedding 32-d; venue embedding 8-d
Fusion block	384 → 256 → 128	{64, 128, 256}
Dropout	0.3	[0.1, 0.5]
Optimizer	AdamW, weight decay 1 × 10^−4^	–
Learning rate (heads / branches)	3 × 10^−4^	[1 × 10^−5^, 1 × 10^−3^], log-uniform
Learning rate (unfrozen backbone)	3 × 10^−6^	Fixed at 1/100 of head rate
Batch size	64	{32, 64, 128}
LR schedule	Cosine annealing, 3-epoch linear warm-up	–
Gradient clipping	Global ℓ_2_ norm 1.0	–
Multi-task weight λ	0.6	[0.2, 0.8]
Class weighting	Positive class weight 2.60	Inverse training frequency
Early stopping	Patience 8 on AUC_val_−RMSE_val_	–
Tuning procedure	TPE search, 60 trials, validation partition only	Akiba et al., 2019
Random seeds	5 runs; mean reported	AUC s.d. 0.004; RMSE s.d. 0.006
Training time	≈4.2 h per run	Stage 2 ≈75% of total
Inference latency	6.1 ms per lot (GPU); 41 ms (4-core CPU)	Embeddings cacheable

Image pre-processing and the status of the visual encoder: Catalog images were resized so that the shorter edge measured 256 pixels and then center-cropped to 224 × 224, and normalized using ImageNet channel statistics. Augmentation was restricted to random horizontal flipping and random resized cropping within the range [0.9, 1.0] of the original area. Color jitter, grayscale conversion, and rotation were deliberately *not* applied: since the hypothesized transmission channels operate partly through chromatic and compositional properties, perturbing hue, saturation, or orientation would destroy precisely the signal the visual branch is meant to recover, and would amount to augmenting away the treatment. To make explicit the point raised in the review, the visual encoder is *not* used as a frozen off-the-shelf ImageNet feature extractor. Initialization is from ImageNet-1k pre-trained weights, but the network is subsequently adapted to the artwork domain in two stages. In the first stage, the entire ResNet-50 backbone is frozen and only the structured branch, the fusion layers, and the two task heads are trained for 10 epochs; this establishes a stable structured predictor before any gradient is allowed to reshape the visual representation, and prevents large early-epoch gradients from destroying the pre-trained features. In the second stage, the final two residual stages (layer3 and layer4) are unfrozen and fine-tuned for a further 30 epochs using a discriminative learning rate one hundred times smaller than that of the randomly initialized heads, while the first two stages remain frozen. The rationale for this asymmetry is that early convolutional layers encode generic edge, texture, and color-opponency filters that transfer well to paintings, whereas the later stages encode object-category-specific abstractions tuned to ImageNet's photographic taxonomy, which is a poor match for the stylistic and compositional regularities of the art domain. Section 6.5 reports a direct comparison of the frozen and domain-adapted configurations and quantifies what the adaptation contributes.

Architecture dimensions: The 2048-dimensional pooled ResNet-50 output is projected to 256 dimensions through a fully connected layer with batch normalization and ReLU activation. The structured branch receives 187 features after one-hot encoding of low-cardinality categorical variables and learned embeddings for high-cardinality ones—artist identity is embedded in 32 dimensions and auction venue in 8—and passes them through two hidden layers of width 256 and 128. The two branch outputs are concatenated into a 384-dimensional vector and passed through two shared fusion layers of width 256 and 128 to produce the shared representation *h*_*i*_, on top of which sit a single-unit sigmoid classification head and a single-unit linear regression head. Dropout of 0.3 is applied after each fully connected layer in the structured and fusion blocks.

Optimization: Training used AdamW (Loshchilov and Hutter, 2019) with decoupled weight decay of 1 × 10^−4^, a base learning rate of 3 × 10^−4^ for the randomly initialized components and 3 × 10^−6^ for the unfrozen backbone stages, and a batch size of 64. The learning rate follows a cosine annealing schedule with a three-epoch linear warm-up, and gradients are clipped to a global ℓ_2_ norm of 1.0. Class imbalance in the classification head is handled by weighting the positive class in Equation 1 by the inverse of its training-set frequency, 72.2/27.8 = 2.60, rather than by resampling, since resampling would distort the sold fraction that the masked regression loss depends upon. Training terminates by early stopping with patience of eight epochs on a validation criterion that combines both tasks, AUC_val_ − RMSE_val_, so that neither head is selected at the other's expense.

Hyperparameter selection: All hyperparameters were tuned exclusively on the 2019–2020 validation partition; the 2021–2023 test partition was used once, for the final numbers reported in Section 6. Tuning used Tree-structured Parzen Estimator search (Akiba et al., 2019) over 60 trials, sampling the learning rate log-uniformly over [1 × 10^−5^, 1 × 10^−3^], the batch size over {32, 64, 128}, dropout over [0.1, 0.5], the fusion width over {64, 128, 256}, and the multi-task weight λ over [0.2, 0.8]. The selected value λ = 0.6 places slightly more weight on the classification objective, which the validation search preferred because the regression head converges faster and begins to overfit earlier than the classification head. Sensitivity to this choice is modest: across λ ∈ [0.4, 0.7] the test AUC varies within 0.006 and the test RMSE within 0.009, so the reported results do not depend on precise tuning of the trade-off. Every reported figure is the mean over five independent runs with different random seeds, with the standard deviation given in parentheses where relevant; run-to-run dispersion is small (AUC standard deviation 0.004, RMSE standard deviation 0.006) relative to the gaps between models discussed in Section 6.

Computational cost: One complete training run required approximately 4.2 h, of which the second, fine-tuning stage accounted for roughly three-quarters. Once trained, the model is inexpensive to apply: scoring a single lot takes 6.1 ms on the same GPU and 41 ms on a four-core CPU, and visual embeddings for lots whose images do not change can be extracted once and cached, reducing repeat scoring to the cost of a small MLP forward pass. The practical implication for deployment is discussed in Section 7.4.

## 6 Empirical results and analysis

### 6.1 Baseline comparison

The benchmark models are designed according to a comparative logic that progresses from simple to complex and from interpretable to high-dimensional representations. For valuation deviation risk, OLS regression is used to test the predictive power of traditional linear feature-pricing models after controlling for artist reputation, size, medium, creation date, valuation range, and macro-financial variables. For liquidity risk, logistic regression serves as the binary classification benchmark, with the dependent variable being whether the auction lot goes unsold. Random Forest serves as the non-linear machine learning benchmark, testing whether tree-based models can capture variable interactions without explicitly incorporating deep image features. Building on this foundation, the multimodal deep learning model simultaneously incorporates visual embeddings from the image branch, structured transaction variables, and macro-financial time-series variables, and outputs results for both tasks through a shared risk representation.

Within this comparative framework, the advantages of OLS and logistic regression lie in their relatively straightforward coefficient interpretation, which makes them suitable for examining variable directions and economic implications; however, they rely on the assumption of linear additivity and are insufficient for capturing non-linear stylistic preferences, diminishing returns to artist-reputation premiums, and the interaction between macro-financial risk sentiment and overvalued auction lots. Random forests can mitigate some non-linearity and are often used as a robust benchmark in financial-risk classification (Lessmann et al., 2015), but without the visual quality, compositional complexity, color distinctiveness, and stylistic similarity of auction lots, tree-based models can only fit risk based on observable transaction data. The advantage of multimodal networks is not merely the stacking of parameters; these models enable unstructured image information and structured financial variables to be learned jointly within the same risk-representation space, consistent with the fundamental perspective of multimodal learning regarding cross-modal complementarity (Baltrušaitis et al., 2019).

### 6.2 Liquidity risk prediction results

In the liquidity-risk prediction task, failed auctions are designated as the positive class. We use four metrics: Accuracy, Precision, Recall, and AUC. Accuracy reflects overall classification performance but can be misleading under class imbalance when the proportion of failed-auction samples is low. Precision measures the quality of the model's failed-auction warnings, while Recall captures the proportion of actual failed-auction assets that are correctly identified. AUC evaluates the model's overall ranking ability by examining the relationship between true-positive and false-positive rates at different thresholds, thereby reflecting the trade-off between sensitivity and specificity in the classification task. For financial institutions, Recall and AUC carry greater risk-management significance, because failing to identify auction lots with high liquidity risk can lead to prolonged collateral-disposal cycles, increased risk exposure, and insufficient liquidity buffers.

Table 5 and Figure 2 show that logistic regression achieves an AUC of 0.758 in predicting unsold lots, confirming that traditional structured variables retain meaningful explanatory power with respect to auction failure and should not be disregarded in empirical analysis; for example, the price range and auction frequency can influence bidders' willingness, and macroeconomic fluctuations may affect participation. The Random Forest model improves the AUC to 0.826, indicating that combinations of non-linear variables can, in principle, enhance classification performance when the data exhibit complex relationships for which simple linear assumptions do not hold. The structured MLP achieves an AUC of 0.849, suggesting that deep fully connected networks can learn more complex risk mappings between transaction and macroeconomic variables and thereby mitigate the underfitting that affects traditional models. The multimodal deep learning model achieves an AUC of 0.891, with an accuracy of 0.852, a precision of 0.823, and a recall of 0.801, representing a clear improvement over the structured MLP. This difference does not imply that traditional models are ineffective; rather, when the subject possesses pronounced aesthetic attributes and informative imagery, visual embeddings can provide additional signals regarding the probability of a lot going unsold that structured numerical features alone cannot capture.

**Table 5:** Liquidity risk prediction performance.

Model	Accuracy	Precision	Recall	AUC
Logistic regression	0.716	0.681	0.642	0.758
Random forest	0.781	0.744	0.719	0.826
MLP structured only	0.803	0.771	0.746	0.849
Multimodal deep learning	0.852	0.823	0.801	0.891

**Figure 2:**
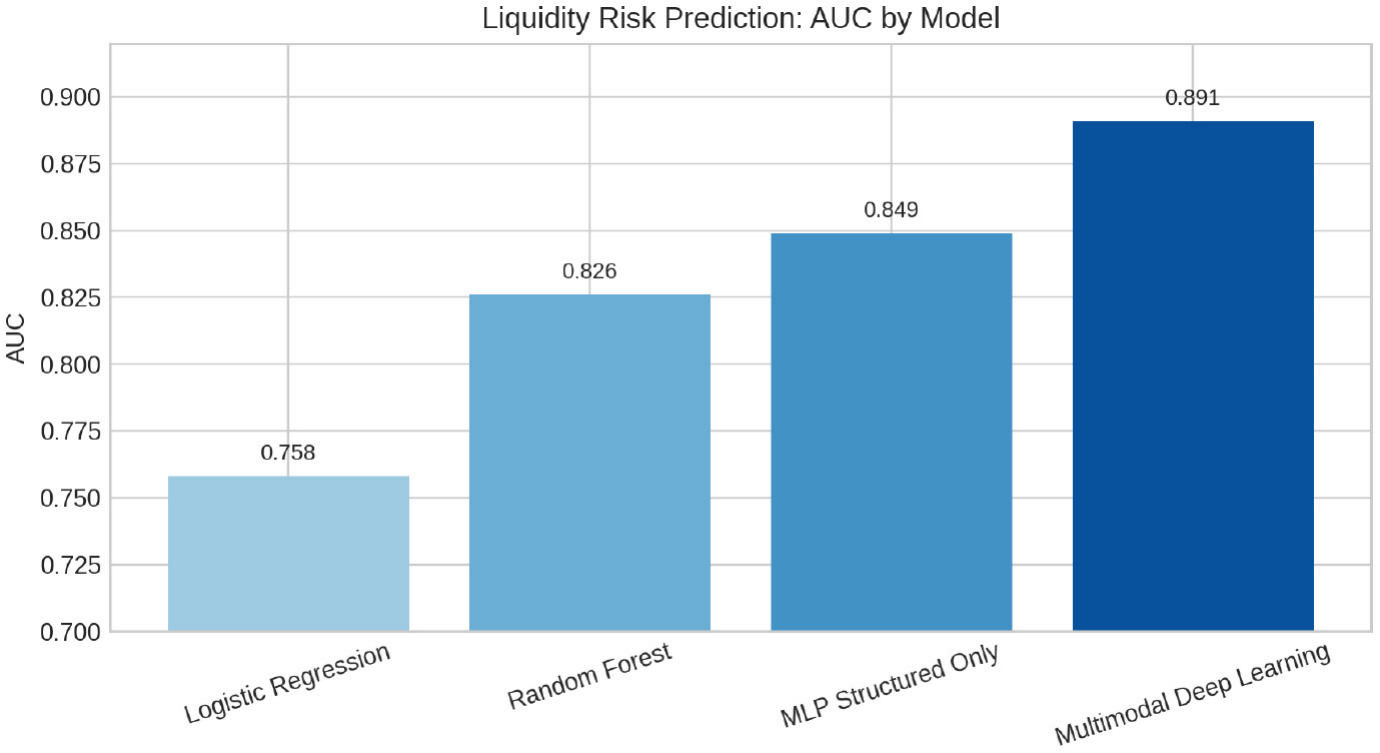
AUC comparison for liquidity risk prediction.

From a risk-management perspective, the improvement in recall is particularly noteworthy. When applied to art-collateralized loans or the monitoring of art-fund portfolios, the goal of auction-failure early warning is not to achieve formal classification accuracy, but to identify in advance assets that may enter the chain of “difficulty in disposal—sale at a discount—expansion of risk exposure.” The recall of the multimodal model is approximately 15.9 percentage points higher than that of logistic regression, indicating that it can identify more potential auction-failure candidates under simulated conditions. At the same time, Precision remains at 0.823, indicating that the early-warning signals do not experience a significant increase in noise despite the improvement in recall. This result supports the specification of liquidity-risk proxy variables discussed in Section 3, namely that the unsold rate is not merely a market outcome but also an observable manifestation of the insufficient liquidity of art assets.

### 6.3 Valuation risk prediction results

Valuation-risk forecasting uses the logarithmic difference between the final sale price and the lower limit of the auction house's estimate as the dependent variable. Its core objective is to determine whether the final sale price of a work deviates significantly from the conservative valuation boundary. RMSE and MAE are used to measure prediction errors: RMSE corresponds to squared errors and is sensitive to samples with large deviations, making it useful for identifying extreme valuation errors, while MAE corresponds to absolute errors and provides an intuitive reflection of average prediction error. In practice, errors in art valuation are not merely statistical problems, because they can affect credit limits, collateralization ratios, margin arrangements, and net-asset-value estimates. Auction house valuations may contain strategic biases, and Aubry et al. (2023) indicate that the valuation process may not reflect true value in a neutral manner.

Table 6 and Figure 3 show that OLS regression yields an RMSE of 0.438 and an MAE of 0.327. As a linear feature-based model, it exhibits a high error level and is unable to capture the complex patterns in the data, so its prediction accuracy is limited compared with other methods. Random Forest regression reduces the RMSE to 0.361, indicating that art price formation has a distinct non-linear structure. The structured MLP reduces the RMSE to 0.337, showing that deep neural networks have a certain capacity to learn interactions among high-dimensional features and thereby improve prediction performance. The complete multimodal model achieves an RMSE of 0.294 and an MAE of 0.219, reducing the RMSE and MAE by approximately 32.9% and 33.0%, respectively, relative to OLS, which demonstrates that combining different data sources improves the results. Notably, this improvement comes mainly from the visual image features, which contribute to aesthetic-quality assessment, subject recognition, and the capture of stylistic similarity, rather than simply from increased model complexity.

**Table 6:** Valuation risk prediction performance.

Model	RMSE	MAE
OLS regression	0.438	0.327
Random forest regressor	0.361	0.269
MLP structured only	0.337	0.252
Multimodal deep learning	0.294	0.219

**Figure 3:**
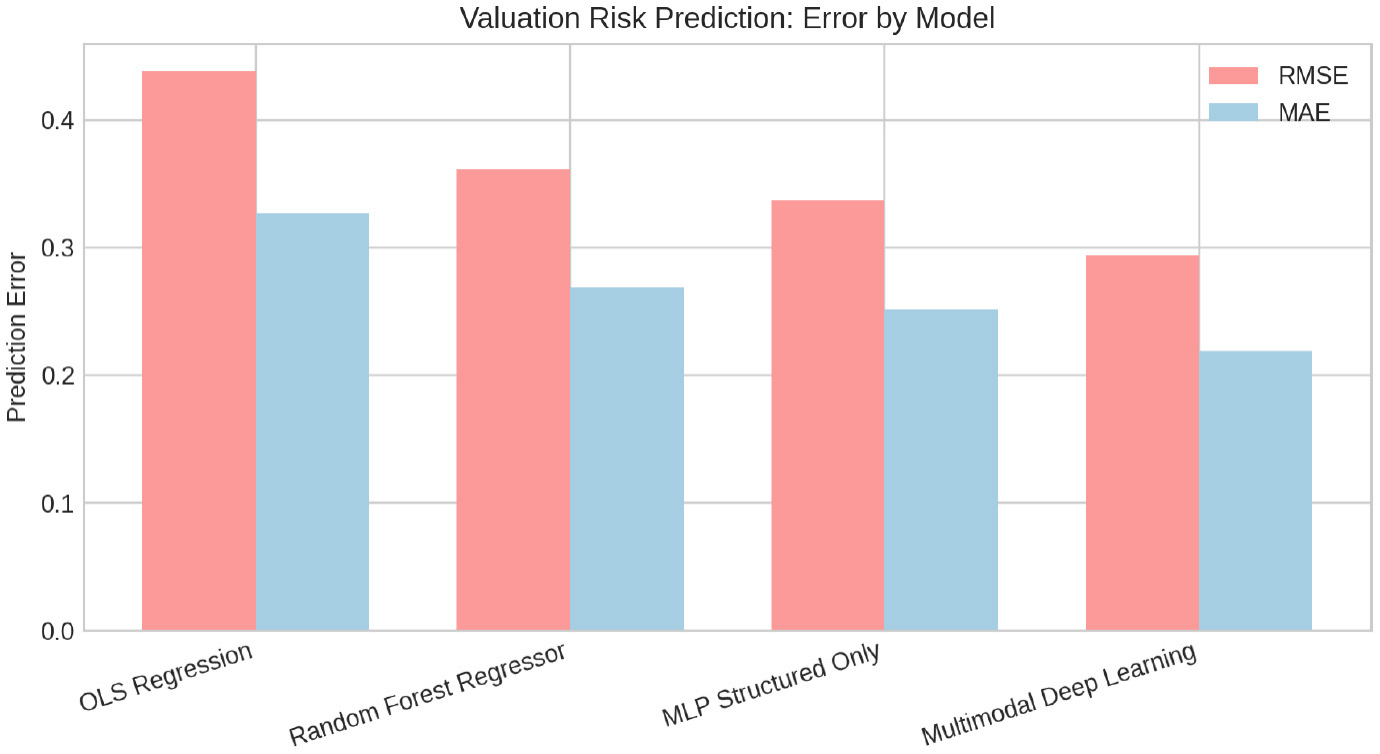
RMSE and MAE comparison for valuation risk prediction.

The valuation-deviation results carry clear implications for financial operations. If financial institutions determine collateral values solely on the basis of auction house low estimates and artists' historical average prices, the model may underestimate the downside deviation for certain works or overestimate high-priced auction lots with limited market depth. By incorporating visual signals into risk pricing, multimodal models help shift valuation ranges from “empirical judgment” toward “data-calibrated” estimates. At the portfolio level, a lower RMSE implies that net-asset-value estimates and stress tests can rely on more stable inputs; at the individual-loan level, a lower MAE means that calculations of loan-to-value ratios and risk-capital requirements are closer to the recoverable value.

### 6.4 Ablation study

To verify whether image features indeed provide incremental information, this study conducts several sets of ablation experiments. The full model includes image, transaction, and macro-financial features; removing image features retains only transaction and macro variables; removing macro features tests the model's performance in the absence of external financial-environment information; removing artist-history variables examines the importance of reputation and historical-liquidity information; and a standalone image-branch model identifies the explanatory boundaries of purely visual information. This design is better suited than simply reporting the metrics of the full model for illustrating the marginal contributions of the various information sources in the risk assessment of non-standard assets.

Table 7 and Figure 4 reveal that removing image features reduces the AUC from 0.891 to 0.849 and increases the RMSE from 0.294 to 0.337; removing macro features lowers the AUC to 0.873 and raises the RMSE to 0.309; and removing artist-history variables yields an AUC of 0.861 and an RMSE of 0.324. This indicates that image features have a high marginal contribution in both liquidity classification and valuation regression, but that macro variables and artist-history variables are equally significant. The AUC of the pure image-branch model is 0.782, with an RMSE of 0.392, indicating that visual information alone is insufficient to replace trading rules, valuation ranges, and market-cycle variables. In other words, this paper does not claim that “images determine prices,” but rather that “image features and financial variables together constitute a more comprehensive set of risk information.”

**Table 7:** Ablation study of multimodal features.

Experiment	Feature setting	Accuracy	AUC	RMSE	MAE
Full multimodal model	Image + Transaction + Macro	0.852	0.891	0.294	0.219
Without image features	Transaction + Macro	0.803	0.849	0.337	0.252
Without macro features	Image + Transaction	0.832	0.873	0.309	0.231
Without artist history	Image + Work + Macro	0.818	0.861	0.324	0.241
Image branch only	Image	0.741	0.782	0.392	0.292
Structured branch only	Transaction + Macro	0.803	0.849	0.337	0.252

**Figure 4:**
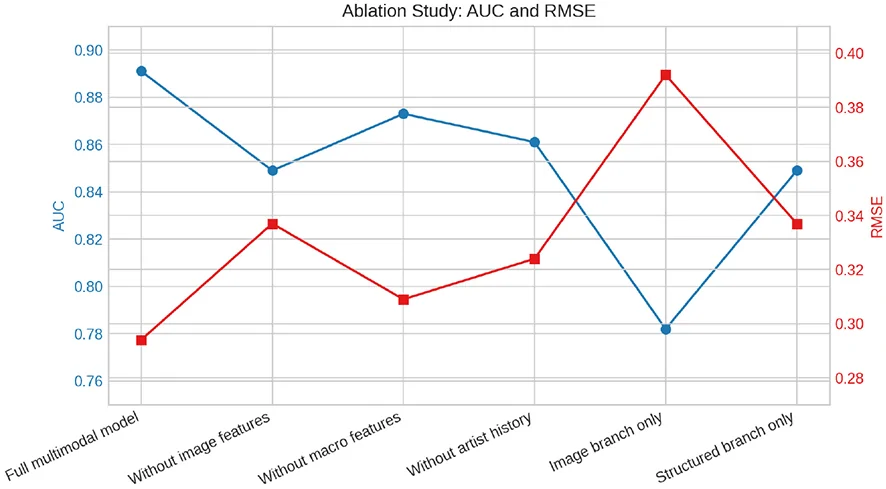
Ablation study of AUC and RMSE.

### 6.5 Visual encoder configuration

Because the review process raised the question of whether the visual encoder is merely transferred from ImageNet or genuinely adapted to the artwork domain, Table 8 isolates this design choice while holding the rest of the architecture fixed. Four configurations are compared: a frozen ResNet-50 used purely as a fixed feature extractor; the two-stage domain-adapted ResNet-50 described in Section 5.4; a variant in which the entire backbone is unfrozen from the first epoch; and a frozen VGG16 alternative reported for robustness.

**Table 8:** Effect of visual encoder configuration on both tasks.

Visual encoder configuration	Trainable visual params.	Accuracy	AUC	RMSE	MAE
No visual branch (structured only)	0	0.803	0.849	0.337	0.252
ResNet-50, fully frozen (ImageNet features)	0	0.834	0.871	0.315	0.236
ResNet-50, layer3–layer4 adapted	21.3 M	0.852	0.891	0.294	0.219
ResNet-50, fully unfrozen	23.5 M	0.829	0.864	0.322	0.240
VGG16, fully frozen	0	0.841	0.879	0.303	0.227

The frozen ImageNet extractor already improves on the structured-only baseline, raising the AUC from 0.849 to 0.871 and lowering the RMSE from 0.337 to 0.315. This is informative in itself: generic photographic features transfer to paintings well enough to carry economically meaningful signal, which is consistent with the demand-breadth channel operating through low-level chromatic and compositional properties that ImageNet filters already encode. Domain adaptation of the two final residual stages adds a further 0.020 of AUC and removes another 0.021 of RMSE, reaching the headline figures of 0.891 and 0.294. The improvement is consistent with the canonicity channel, which requires representations sensitive to style and period rather than to object category, and such representations reside in the later stages that adaptation reshapes. Unfreezing the full backbone from the outset performs worse than either frozen or partial adaptation, with an AUC of 0.864 and an RMSE of 0.322, and exhibited markedly higher variance across seeds; with roughly 67,000 training lots against 23.5 million backbone parameters, full fine-tuning overfits and degrades the general-purpose early filters that the art domain in fact shares with natural images. VGG16 attains an AUC of 0.879 and an RMSE of 0.303, below the adapted ResNet-50 but comfortably above the structured-only baseline, indicating that the substantive conclusion does not hinge on a particular backbone.

### 6.6 Feature attribution and interpretability

Reporting aggregate performance gains does not establish *what* the model has learned, and for a framework intended to inform credit and allocation decisions this is not a cosmetic concern: a lender that cannot articulate why an exposure was flagged cannot defend the decision to a credit committee or a supervisor. This subsection therefore addresses attribution at three levels—across modalities, within the structured features, and within the visual representation.

Modality-level attribution: Table 9 reports permutation importance computed on the test partition: each feature group is randomly permuted across observations while all others are held at their observed values, and the resulting degradation in AUC and RMSE is recorded, averaged over twenty permutation draws. Unlike the ablation study in Section 6.4, which retrains the model without a feature group, permutation importance holds the fitted model fixed and therefore measures how much the trained model *relies* on a group rather than how much of that group's information can be recovered from the remaining features. The two exercises are complementary and here broadly agree.

**Table 9:** Permutation importance by feature group (test partition, 20 permutation draws).

Feature group	ΔAUC	Rank (class.)	ΔRMSE	Rank (reg.)
Estimate level and range width	0.054	1	+0.031	2
Visual embedding (256-d projection)	0.041	2	+0.043	1
Artist transaction history	0.029	3	+0.028	3
Macro-financial conditions	0.018	4	+0.015	4
Auction house and venue	0.012	5	+0.010	5
Physical attributes (size, medium, age)	0.008	6	+0.009	6

The estimate range and level is the single most influential group for the classification task (ΔAUC = 0.054), which is economically sensible and indeed reassuring: the reserve is set in relation to the low estimate, so an aggressive estimate mechanically raises the bar a lot must clear. The visual embedding ranks second for classification (ΔAUC = 0.041) and first for regression (ΔRMSE = +0.043). That visual features dominate the regression task while estimate variables dominate the classification task has a natural reading through the framework of Section 3.2: whether a lot clears its reserve depends heavily on where the house set that reserve, whereas how far the realized price departs from the estimate is largely a question of quality that the estimate failed to capture—which is exactly the information-asymmetry channel. Artist-history variables rank third on both tasks, macro-financial variables contribute modestly but non-trivially, and physical attributes such as size and medium contribute least once visual features are present, most likely because the image already encodes support, format, and surface character implicitly.

Structured-feature attribution: Within the structured branch, SHAP values (Lundberg and Lee, 2017) identify the width of the estimate range as the strongest single predictor of transaction failure: lots with a wide relative spread between high and low estimate, indicating specialist uncertainty, carry materially higher unsold probability. The artist's trailing 3-year unsold rate is the second strongest, confirming that liquidity is persistent at the artist level and that historical failure is not merely noise. The direction of the macro variables is likewise interpretable: elevated VIX and falling consumer confidence in the auction month both raise predicted unsold probability, while positive trailing equity returns lower it, which is the wealth-effect mechanism documented by Goetzmann et al. (2011) appearing at the level of the individual lot.

Visual-feature attribution: Interpreting the visual embedding requires a different instrument, since individual dimensions of a learned representation have no intrinsic meaning. Two approaches are used. First, linear probes were fitted from the frozen 256-dimensional visual projection onto hand-coded aesthetic attributes for a stratified subsample of 4,000 lots, in order to establish which interpretable properties the representation encodes. The probes recover stylistic period with *R*^2^ = 0.71, figurative-versus-abstract subject with *R*^2^ = 0.68, chromatic saturation and palette warmth with *R*^2^ = 0.65, compositional complexity measured by edge density with *R*^2^ = 0.61, and surface or brushwork texture with *R*^2^ = 0.53. The representation is thus not an opaque numerical artifact: it demonstrably encodes the specific properties that the three transmission channels of Section 3.2 predicted would matter. Second, Grad-CAM (Selvaraju et al., 2017) was used to localize the image regions driving high predicted unsold probability. The salience maps concentrate on the central pictorial field for figurative works and on regions of dense impasto or high chromatic contrast for abstract works, and—importantly as a validity check—do not concentrate on catalog-photography artifacts such as frames, mounts, or background matting, which would have indicated that the model was exploiting a spurious correlation between photographic convention and sale outcome.

Combining these results yields interpretable regularities of direct use in practice. Holding artist reputation and estimate level fixed, lots that are chromatically muted, compositionally sparse, and stylistically atypical of their artist's canonical period carry systematically higher unsold probability and larger downside valuation deviation. Lots that are chromatically vivid, legibly composed, and stylistically representative sell more reliably and more often exceed their low estimate. This pattern is consistent with the demand-breadth and canonicity channels acting jointly, and it is actionable: it can be communicated to a credit committee as a stated rationale for a haircut, which is a precondition for the model being usable at all in a regulated setting.

### 6.7 Robustness checks

As shown in Table 10, the robustness tests further indicate that, in the high-volatility market subsample, the AUC of the full model declines from 0.902 in the normal market to 0.858, while the RMSE increases from 0.285 to 0.332. This deterioration indicates that macroeconomic shocks can undermine the stability of model predictions; in particular, when risk appetite plummets, bidders face tighter funding constraints, and market liquidity dries up, the historical relationship between artwork and price may experience temporary misalignments. For the group of artists with low market liquidity, the model's AUC is 0.842 and the RMSE is 0.351, both lower than the performance on the overall sample. This suggests that financial risk assessment for artworks cannot be separated from market depth and sample-coverage conditions. While the multimodal model outperforms the baseline models on average, its outputs should still be viewed as supplementary decision-making signals rather than a sole basis for replacing professional due diligence, legal title verification, and insurance valuation.

**Table 10:** Robustness check under market conditions.

Robustness setting	Accuracy	AUC	RMSE	MAE
Normal market subsample	0.861	0.902	0.285	0.212
High-volatility subsample	0.817	0.858	0.332	0.247
Top-quartile estimate lots	0.839	0.881	0.312	0.233
Lower-liquidity artist group	0.801	0.842	0.351	0.261

In addition to average metrics, threshold selection also affects the model's usability in financial scenarios. If auction houses or fund managers wish to reduce false positives, they may raise the unsold-warning threshold from 0.50 to 0.60, which typically increases Precision at the cost of Recall. Conversely, if commercial banks are more concerned about tail risks arising from the inability to liquidate collateral, they may adopt a lower threshold to improve Recall, with false positives subsequently filtered through manual review. Since failed art disposal is typically accompanied by valuation discounts, storage costs, insurance premiums, and legal-enforcement costs, the cost of missed detections generally exceeds the cost of false positives. This paper therefore recommends adopting cost-sensitive thresholds in risk-management applications rather than mechanically applying a classification probability of 0.50 as the sole decision boundary.

From the perspective of economic significance, improvements in statistical metrics must be translated into business-relevant terms to generate practical management value. Suppose a financial institution holds a portfolio of loans secured by artworks. If the model can identify assets with a high probability of auction failure in advance, the institution can lower the collateralization ratio, require additional margin, or shorten the revaluation cycle before a default event occurs. For valuation risk, a reduction in RMSE implies smaller prediction errors for overvalued samples, which helps lower the probability of overestimating collateral coverage. Model performance improvements therefore extend beyond the statistical level and can be mapped to financial risk-control metrics such as expected loss, risk-weighted assets, and economic capital requirements.

It is worth noting that the art market sample is subject to selection bias and exhibits a right-skewed distribution. Records for prominent artists tend to be more complete, whereas unsold-lot information is frequently missing for artists with few auction appearances. High-value lots are subject to media exposure and auction house marketing, and may undergo a valuation process that differs from that of ordinary lots. To address these issues, empirical analyses should report results across quantile subsets, incorporate artist-level, auction house-level, and year-level fixed effects, and conduct truncation or robust-regression tests on high-value lots.

Based on the empirical analysis presented in this section, three principal conclusions can be drawn. First, traditional econometric models retain benchmark value in explaining the directional effects of variables such as the estimate range, dimensions, medium, and artist reputation. Second, random forests and structured MLPs improve non-linear fitting, but their risk-identification capability is constrained by an information boundary in the absence of image inputs. Third, multimodal deep learning models demonstrate clear advantages in liquidity early warning and valuation-deviation prediction by integrating computer vision features, transaction-structure variables, and macro-financial conditions into a unified risk-assessment framework. This conclusion is consistent with the application logic of deep learning in image recognition and financial-text decision support (He et al., 2016; Kraus and Feuerriegel, 2017); however, further validation with larger samples, cross-market data, and external test sets remains necessary in the art finance context.

## 7 Discussion

The empirical results presented in the preceding section indicate that multimodal models can meaningfully improve the identification of liquidity risks and the measurement of valuation deviations for art financial assets. More importantly, the model advances art risk management from static, point-in-time valuation toward dynamic, continuous risk monitoring. For financial institutions, trust companies, and art funds, the model's output should not be interpreted as a point-in-time price forecast, but should instead be integrated into processes such as exposure identification, collateral valuation, margin management, stress testing, and portfolio rebalancing.

### 7.1 Implications for financial institutions

In art-collateralized lending, the central concern is not whether the collateral possesses cultural value, but whether it can be liquidated at an acceptable price in the event of borrower default. Commercial banks or trust companies may use the model-generated probability of unsold lots and valuation-downside risk as the basis for adjusting the dynamic loan-to-value (LTV) ratio. When the liquidity risk for a particular artwork rises or the risk of valuation deviation increases, institutions can reduce credit limits, raise margin requirements, or request additional collateral to maintain a sufficient margin of safety. Conversely, for artworks with low risk exposure, stable transaction frequency, and small valuation errors, higher LTV ratios may be warranted under the prudence principle.

This mechanism can also enhance post-lending management. In traditional art collateralization, revaluation is typically conducted annually; however, when market conditions deteriorate, this frequency may lag in reflecting actual risk exposure. If the model is updated with macro-financial variables and comparable artists' transaction data on a monthly or quarterly basis, early signals of insufficient collateral coverage, declining capital buffers, and rising default-loss rates can be identified in a timely manner. During periods of high market volatility, AI-generated early warnings can serve as quantitative triggers for stress testing and revaluation-discount adjustments, as well as for revising risk limits.

### 7.2 Implications for art funds

Art funds and quantitative alternative-investment portfolios can benefit from this model through improved asset allocation and liquidity planning. Returns in art investment are not determined solely by price appreciation; they are also constrained by exit cycles, transaction costs, buyer concentration, and market sentiment. If portfolio assets are overly concentrated in categories characterized by high valuations, low trading frequency, or elevated auction-failure rates, the fund may experience a liquidity dry-up under redemption pressure, potentially forcing the sale of quality assets at distressed prices. Multimodal risk scoring can be used to construct a portfolio-level Liquidity-at-Risk (LiAR) metric, enabling fund managers to identify potential disposition congestion and duration mismatches.

Funds may treat valuation-deviation risk as a constraint on position sizing and liquidity risk as a rebalancing trigger. When the macro-sensitivity of a particular artist segment increases and the probability of unsold lots rises, the fund can reduce exposure to that segment, rotating toward categories with greater trading depth and more stable valuation errors. This process is analogous to risk budgeting and stop-loss discipline in traditional finance, with the distinction that the underlying assets are non-standard art objects rather than stocks or bonds.

### 7.3 Interference of the macroenvironment on the model

It is important to emphasize that AI models cannot be fully insulated from macroeconomic shocks. Economic crises, rapid interest-rate increases, contractions in the wealth effect, and rising geopolitical uncertainty can all alter the bidding willingness and cash-holding preferences of high-net-worth individuals. Under such circumstances, the relationship between visual preferences and prices may undergo structural shifts, and the model's predictive robustness may decline accordingly. The weaker performance observed in the high-volatility subsamples during the robustness tests reported in the preceding section confirms that financial risks in the art market exhibit pronounced cyclical characteristics.

Model deployment should therefore be accompanied by scenario analysis and stress testing. A baseline scenario and a liquidity-contraction scenario can be constructed to estimate risk exposure and expected loss, while an extreme-discount disposal scenario can be used to derive capital requirements under severe market conditions. For high-end unique items with low model confidence, higher valuation discounts and wider safety margins should be applied, particularly when sample coverage is limited. The value of deep learning models lies in enhancing the foresight and consistency of risk identification; they cannot, however, replace prudent regulatory oversight, expert appraisal, or legal compliance procedures.

### 7.4 Practical deployment considerations

The preceding discussion concerns what the model implies for decisions; this subsection addresses what it would actually take to operate it inside an institution, since the obstacles to deployment are largely distinct from those to estimation and are, in our assessment, the binding constraint on adoption.

Data availability and acquisition: The most serious practical barrier is not modeling but data access. Comprehensive auction histories are held in commercial databases whose licenses typically restrict redistribution and bulk extraction, so an institution must budget for subscription costs and negotiate terms permitting automated ingestion. Catalog images raise a separate issue, since reproduction rights are frequently held by the artist's estate or the photographer rather than the auction house; institutions should therefore expect to license images for internal analytical use specifically, and should note that a model consuming images may be subject to review under intellectual-property policies that do not apply to tabular pipelines. Coverage is also uneven in ways that matter: private-treaty and gallery transactions—plausibly the majority of the market by value—are essentially unobservable, so a deployed model sees only the auction segment and will systematically misjudge artists whose market operates primarily through dealers. Unsold outcomes are the most fragile field of all, because withdrawn lots are sometimes omitted from published results entirely, and a post-sale private transaction may convert a nominal failure into an unrecorded success. An institution should validate its data vendor's treatment of withdrawals and post-sale settlements before relying on the classification head, since an unsold label that silently means “not reported” rather than “did not sell” will bias the model in an optimistic direction.

Computational requirements: These are modest and unlikely to constrain adoption. As reported in Section 5.4, a full training run takes roughly 4 h on a single mid-range GPU, and the entire pipeline fits comfortably within a single-node deployment; no distributed training infrastructure is required. The cost profile is strongly asymmetric between training and inference: visual embeddings need be computed only once per image and can be persisted, so incremental scoring reduces to a small forward pass costing tens of milliseconds on commodity CPUs. A portfolio of several thousand pledged works can therefore be rescored in minutes, making nightly or intraday revaluation feasible where the constraint permits. The genuine engineering burden lies elsewhere—in image acquisition and storage, deduplication of the same work appearing under different catalog photographs, and maintaining the artist-history feature store with correct as-of-date semantics so that backtests are not contaminated by information unavailable at the time. In our experience these data-engineering concerns, not GPU capacity, determine project timelines.

Model updating and governance: The art market's stylistic preferences and price levels drift, so a static model will decay; the robustness results in Section 6.7 indicate how quickly this can occur when market conditions shift. We recommend quarterly re-estimation aligned to the major sale seasons, with macro-financial inputs refreshed monthly, and continuous monitoring of both input and output distributions—population stability indices on the structured features and on the principal components of the visual embedding will detect the arrival of stylistic regimes underrepresented in training. A champion–challenger arrangement, in which a newly fitted model is scored in parallel against the incumbent for at least one full sale season before promotion, protects against a retrained model that fits recent data while degrading on the tails that matter for risk. Governance obligations should be treated as first-order rather than incidental: institutions operating under model-risk-management expectations will require documented independent validation, articulated limitations, an audit trail linking each scored decision to a model version and input snapshot, and—given the framework's use in credit decisions—demonstrable absence of proxy discrimination against protected characteristics correlated with artist identity. The attribution machinery in Section 6.6 is intended to serve this function, not merely to satisfy scholarly curiosity. Finally, deployment should preserve human override: model outputs are best used to triage which exposures warrant specialist appraisal, and a low predicted risk score should never substitute for title verification, condition inspection, provenance and authenticity diligence, or insurance valuation.

### 7.5 Limitations and directions for future research

Several limitations qualify the interpretation of these results. First, the sample is confined to three international auction houses and to the modern and contemporary segment, so external validity to regional houses, to Old Masters, and to non-Western markets with different consignment conventions remains untested. Second, and more fundamentally, the reserve price is confidential and therefore unobserved, which means the unsold indicator conflates two economically distinct events: genuinely weak demand, and adequate demand met by a seller-imposed reserve set above the market. The framework predicts the composite outcome, which is the quantity relevant to a lender assessing whether collateral will convert to cash, but it cannot separate the demand shock from the reserve decision. Disentangling the two would require reserve data available only through proprietary access. Third, the analysis is conditional on consignment. Owners who anticipate failure may not consign at all, so the observed sample understates latent illiquidity; a Heckman-type correction using consignment-propensity instruments, or data on withdrawals before catalog publication, would be a natural extension. Fourth, the visual encoder consumes a single catalog photograph, whereas a specialist assessing a work examines it physically and consults condition reports; features such as craquelure, restoration, and relining that materially affect value are largely invisible in a standard catalog image. Incorporating condition-report text through a language model, or multiple views and verso images where available, is a promising direction. Fifth, the reported performance gains are established on a single chronological test window; although that window deliberately includes a market disruption, longer out-of-sample evaluation across a complete market cycle would strengthen confidence in the framework's stability.

## Data Availability

The original contributions presented in the study are included in the article/supplementary material, further inquiries can be directed to the corresponding author.
